# Association Between Preoperative Core Temperature and Postoperative Mortality in Patients with Major Burns

**DOI:** 10.3390/jcm15103785

**Published:** 2026-05-14

**Authors:** Jihion Yu, Young Joo Seo, Hee Yeong Kim, Young-Kug Kim

**Affiliations:** 1 Department of Anesthesiology and Pain Medicine, Asan Medical Center, University of Ulsan College of Medicine, Seoul 05505, Republic of Korea; jihionyu@amc.seoul.kr (J.Y.); umelly98@gmail.com (H.Y.K.); 2 Department of Anesthesiology and Pain Medicine, Hallym University Hangang Sacred Heart Hospital, Seoul 07247, Republic of Korea

**Keywords:** preoperative core temperature, major burns, mortality

## Abstract

**Background and Objectives**: Major burn injury causes profound hypermetabolism and altered thermoregulation. While perioperative hypothermia is linked to adverse outcomes, the prognostic significance of preoperative core temperature in major burn patients remains poorly defined. Therefore, we investigated the association between preoperative core temperature and postoperative mortality in patients with major burns. **Materials and Methods**: This retrospective study included 635 adult patients with major burns who underwent surgery. Preoperative core temperature was measured in the intensive care unit before surgery. The primary outcome was 90-day postoperative mortality. Secondary outcomes were 30-day postoperative complications, including major adverse cardiovascular events (MACE), bloodstream infection, and continuous renal replacement therapy (CRRT) requirement. Cox proportional hazards regression, receiver operating characteristic (ROC) curve, Kaplan–Meier survival, and restricted cubic spline analyses were performed. **Results**: The 90-day postoperative mortality rate was 35.6%. Mortality increased in a graded manner as preoperative core temperature decreased. In multivariable Cox regression analysis, preoperative core temperature remained independently associated with 90-day mortality. Restricted cubic spline analysis showed an inverse linear association between preoperative core temperature and mortality risk. ROC curve analysis identified 37.0 °C as an exploratory and hypothesis-generating cohort-specific threshold for risk stratification. Regarding secondary outcomes, the core temperature ≤37.0 °C group had higher rates of MACE, bloodstream infections, and CRRT requirement (all *p* < 0.05). **Conclusions**: Lower preoperative core temperature was associated with increased 90-day postoperative mortality in adults with major burns undergoing surgery. Preoperative temperature may serve as a clinically relevant marker of physiologic vulnerability and postoperative risk.

## 1. Introduction

Major burn injury induces a profound hypermetabolic and catabolic response, resulting in multiorgan dysfunction and increased susceptibility to infection [1]. Extensive skin loss, increased evaporative heat loss, large-volume fluid resuscitation, and prolonged wound exposure disrupt normal thermoregulation in these patients [2]. Consequently, temperature management is considered an important component of supportive care in major burn patients [3].

Burn patients exhibit sustained hypermetabolism and burn-specific thermodysregulation, and their baseline core temperature is often elevated even in the absence of overt infection [4,5]. This upward shift complicates the interpretation of perioperative body temperature values and creates uncertainty regarding the appropriate preoperative temperature range in patients with major burns [2]. Notably, despite clinical emphasis on temperature management, the clinically appropriate perioperative temperature range remains poorly defined and highly variable across burn centers, even when international guidelines are applied [6,7]. Importantly, perioperative hypothermia has been associated with adverse outcomes, including cardiovascular events, coagulopathy, increased blood loss, and delayed wound healing [8,9,10]. In burn surgery, most preventive strategies have focused on intraoperative temperature management [3,11]. However, the prognostic significance of preoperative core temperature in major burn patients has not been adequately evaluated.

Therefore, this study aimed to investigate the association between preoperative core temperature and 90-day postoperative mortality in patients with major burns. In addition, we evaluated whether preoperative core temperature was associated with 30-day postoperative complications, including major adverse cardiovascular events (MACE), bloodstream infection, and the need for continuous renal replacement therapy (CRRT).

## 2. Materials and Methods

### 2.1. Patients

This retrospective observational study included adult patients with major burns who were admitted to the burn intensive care unit (ICU) at our institution between January 2012 and December 2021. The study protocol was approved by the Institutional Review Board, and the requirement for informed consent was waived owing to the retrospective nature of the study. Adult patients (≥18 years) with major burns, defined as burns involving ≥30% of total body surface area (TBSA), who underwent surgical treatment, were eligible for inclusion. Because the primary objective was to evaluate the prognostic significance of preoperative core temperature during the early post-burn surgical period, patients were excluded if they had incomplete medical records or were lost to follow-up, were transferred after undergoing initial burn surgery at another institution, had documented positive blood cultures prior to surgery, or underwent surgery more than 7 days after burn injury. These criteria were applied to reduce heterogeneity related to prior interventions, preexisting systemic infection, or delayed operative timing. For patients who underwent multiple staged operations, only data from the first surgical procedure were analyzed.

### 2.2. Burn and Temperature Care in ICU

All burn patients admitted to the ICU received initial fluid resuscitation according to a Parkland formula (4 mL/kg/%TBSA), with additional fluids administered to maintain urine output >0.5 mL/kg/h. Burn wound care was performed daily using hydrofoam (Genewel Co., Ltd., Seongnam, Republic of Korea) dressings and topical antimicrobial agents.

Core temperature was continuously monitored throughout ICU admission using a temperature-sensing indwelling urinary catheter (Sewoon Medical Co., Ltd., Cheonan, Republic of Korea), which was routinely used in all major burn patients during the study period. Temperature values were documented at least hourly in the ICU and immediately before transfer to the operating room as part of standard vital sign assessment. For the present study, the preoperative core temperature was defined as the last recorded bladder temperature immediately prior to operating room transfer. The institutional target temperature range was 36.0–38.5 °C. Active warming measures, including forced-air warming blankets (Bair Hugger™, 3M, St. Paul, MN, USA) and fluid warmers (Hotline^®^ fluid warmer, ICU Medical, San Clemente, CA, USA), were applied when the temperature fell below the target range.

### 2.3. Anesthetic Technique

General anesthesia was induced with propofol (Fresofol^®^, Fresenius Kabi Korea, Seoul, Republic of Korea) and rocuronium (Esmeron^®^, Merck Sharp & Dohme, Haarlem, The Netherlands) and maintained with desflurane or sevoflurane according to institutional practice [12,13]. Balanced crystalloid solution was administered at 6–10 mL/kg/h, and red blood cell transfusion was performed when hemoglobin levels were <8 g/dL.

Intraoperative core temperature was continuously monitored using a standard temperature probe after induction of anesthesia and recorded at regular intervals throughout surgery. Active warming measures, including forced-air warming blankets and warmed intravenous fluids, were routinely applied from the beginning of anesthesia to maintain core temperature ≥36 °C. Ambient operating room temperature was maintained between 28 °C and 32 °C. Patient transfer time from the ICU to the operating room was minimized whenever feasible to reduce additional heat loss during transport.

### 2.4. Surgical Technique

Early excision of burn eschar and wound closure were performed according to institutional burn surgery protocols [12,13]. Escharotomy was conducted when circumferential burns were present to relieve tissue pressure, and wound coverage was achieved using split-thickness skin grafts or temporary allografts, depending on wound condition and graft availability. For patients requiring staged procedures, initial coverage with allograft was followed by definitive autologous skin grafting [12].

### 2.5. Data Collection

Demographic and preoperative variables were collected, including age, sex, body mass index, American Society of Anesthesiologists (ASA) physical status, and comorbidities (diabetes mellitus, hypertension, ischemic heart disease, congestive heart failure, and cerebrovascular disease). Burn-related variables included TBSA burned, presence of inhalation injury, mechanism of burn injury, and time interval from burn injury to surgery. Burn size and depth were assessed by experienced burn surgeons according to institutional protocols [14]. The presence of inhalation injury was determined based on clinical findings, history of burn in an enclosed space, bronchoscopic findings (airway edema, soot deposition, mucosal necrosis, or ulceration), and elevated carboxyhemoglobin levels.

Preoperative laboratory data obtained within 24 h before surgery included hemoglobin concentration, white blood cell count, platelet count, serum albumin, serum creatinine, and serum lactic acid levels. Preoperative core temperature was defined as the last bladder temperature recorded in the ICU immediately before transfer to the operating room using a temperature-sensing indwelling urinary catheter and was analyzed as a single value. Intraoperative variables included operation time, vasopressor use, crystalloid and colloid administration, red blood cell transfusion rate and volume.

The primary outcome was 90-day postoperative mortality. Secondary outcomes were 30-day postoperative complications, including MACE, bloodstream infection, and CRRT requirement. MACE included acute myocardial infarction, acute heart failure requiring treatment, clinically significant arrhythmia requiring medication or cardiology consultation, or nonfatal cardiac arrest. Bloodstream infection was defined as clinically treated bacteremia with positive blood cultures during hospitalization. All outcomes were identified through electronic medical record review.

### 2.6. Statistical Analysis

Continuous variables were tested for normality using the Shapiro–Wilk test and are presented as median (interquartile range). Categorical variables are presented as the number of patients (percentage). Continuous variables were compared using the Mann–Whitney U test, and categorical variables were compared using the χ^2^ test or Fisher’s exact test, as appropriate.

Cox proportional hazards regression models were performed to identify independent predictors of 90-day postoperative mortality. To address potential confounding by overall burn-related severity and physiologic status, covariates for the multivariable Cox proportional hazards model were selected a priori based on established burn mortality literature, in addition to baseline group differences and variables with univariable *p* < 0.10. The proportional hazards assumption was evaluated using Schoenfeld residuals. Potential multicollinearity among severity-related covariates was assessed using variance inflation factors. Internal validation of the multivariable Cox model was performed using bootstrap resampling with 200 repetitions. Patients with missing values in variables required for multivariable analysis were excluded using complete-case analysis.

Restricted cubic spline analysis based on the Cox model was performed to explore the continuous association between preoperative core temperature and 90-day postoperative mortality. Receiver operating characteristic (ROC) curve analysis was performed to evaluate the discriminatory ability of preoperative core temperature for 90-day postoperative mortality. Kaplan–Meier survival curves were constructed and compared using the log-rank test according to preoperative core temperature categories.

All statistical analyses were performed using SPSS (version 27.0; IBM Corp., Armonk, NY, USA), MedCalc (version 11.3.3.0; MedCalc Software, Mariakerke, Belgium), and R software (version 4.3.0; R Foundation for Statistical Computing, Vienna, Austria). A two-sided *p*-value < 0.05 was considered statistically significant.

## 3. Results

**Figure 1:**
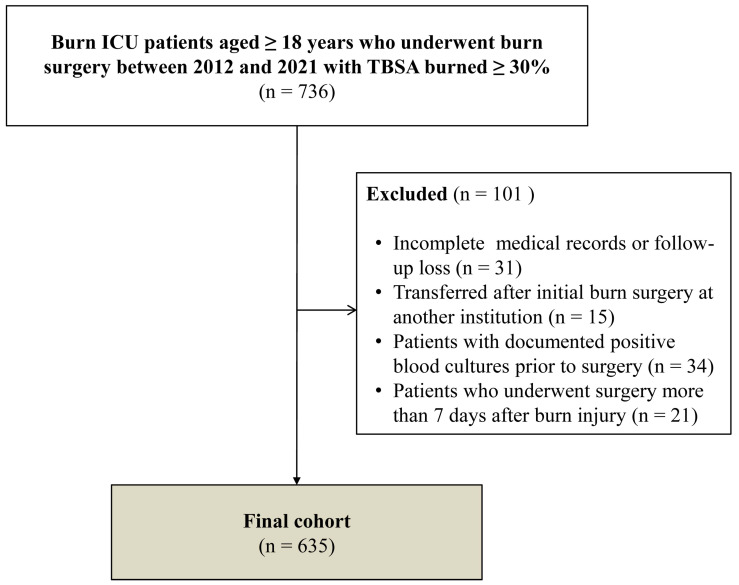
Study cohort flow chart. ICU, intensive care unit; TBSA burned, total body surface area burned.

Of 736 major burn patients admitted during the study period, 635 were included in the final analysis after excluding those with incomplete medical records or loss to follow-up, transfer after initial burn surgery at another institution, documented positive blood cultures prior to surgery, or surgery performed more than 7 days after burn injury (Figure 1).

The overall 90-day postoperative mortality was 35.6%. Baseline characteristics, preoperative laboratory variables, and intraoperative parameters were compared between survivors and non-survivors (Table 1). Compared with survivors, non-survivors were older [54 (46–65) vs. 48 (39–57) years, *p* < 0.001] and had a higher proportion of ASA physical status ≥ 3 [198 (87.6%) vs. 261 (63.8%), *p* < 0.001], diabetes mellitus [32 (14.2%) vs. 25 (6.1%), *p* = 0.001], larger TBSA burned [62.5% (46.8–81.0) vs. 41.0% (34.0–52.0), *p* < 0.001], and more frequent inhalation injury [140 (61.9%) vs. 152 (37.2%), *p* < 0.001]. Although the median time from burn injury to surgery was identical in both groups (3 days), the distribution was slightly shifted toward shorter times in non-survivors, as reflected by the interquartile ranges [3 (2–5) in survivors vs. 3 (2–4) in non-survivors; *p* < 0.001]. Non-survivors also showed greater physiologic derangement, including higher white blood cell count [16.0 (8.6–26.7) vs. 10.8 (6.4–17.1) × 10^3^/μL, *p* < 0.001], lower serum albumin [2.1 (1.8–2.5) vs. 2.4 (2.1–2.7) g/dL, *p* < 0.001], higher serum creatinine [0.9 (0.7–1.2) vs. 0.7 (0.6–0.9) mg/dL, *p* < 0.001], and higher lactic acid levels [4.2 (2.7–6.4) vs. 2.7 (1.8–3.9) mmol/L, *p* < 0.001]. Preoperative core temperature was lower in non-survivors than in survivors [36.8 °C (36.4–37.3) vs. 37.6 °C (37.0–38.0), *p* < 0.001]. Intraoperatively, non-survivors had longer operation time [100 (75–130) vs. 90 (65–120) min, *p* = 0.031], more frequent vasopressor use [149 (65.9%) vs. 151 (36.9%), *p* < 0.001], greater crystalloid administration [21.2 (15.4–31.5) vs. 17.9 (12.3–26.1) mL/kg, *p* < 0.001], and higher RBC transfusion volume [5 (4–8) vs. 4 (3–6) units, *p* < 0.001].

**Figure 2:**
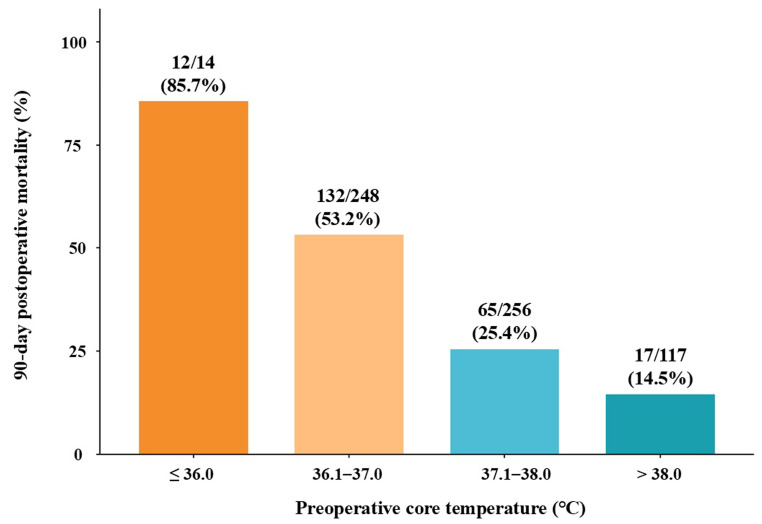
Association between preoperative core temperature and 90-day postoperative mortality. Bars represent mortality rates, with numbers indicating deaths over the total number of patients in each temperature group. The ≤36.0 °C subgroup included a relatively small number of patients; therefore, the corresponding mortality estimate should be interpreted with caution.

Figure 2 demonstrated a stepwise increase in 90-day mortality with decreasing preoperative core temperature. The highest mortality rate was observed in the ≤36.0 °C subgroup (12/14, 85.7%), indicating a markedly elevated risk of death among patients with more pronounced preoperative hypothermia. However, the small sample size warrants cautious interpretation.

In the multivariable Cox proportional hazards model adjusted for clinically relevant covariates and variables associated with mortality in univariable analysis, lower preoperative core temperature remained independently associated with increased 90-day postoperative mortality (adjusted HR, 0.643 per 1 °C increase; 95% CI, 0.513–0.805; *p* < 0.001; Table 2). Other independent predictors included older age, male sex, ASA physical status ≥ 3, diabetes mellitus, larger TBSA burned, inhalation injury, lower serum albumin, higher serum creatinine, and intraoperative vasopressor use. All variance inflation factor values were low (range, 1.09–1.32), indicating no meaningful multicollinearity. The final multivariable model included 16 candidate predictors with 226 mortality events, providing an adequate events-per-variable ratio for model stability. Model assumptions were evaluated, including the proportional hazards assumption, which was tested using Schoenfeld residuals and was not violated (global *p* = 0.140). Internal validation of the multivariable Cox model using bootstrap resampling demonstrated good optimism-corrected discrimination (corrected C-index, 0.839) and acceptable calibration (calibration slope, 0.938), suggesting limited overfitting.

**Figure 3:**
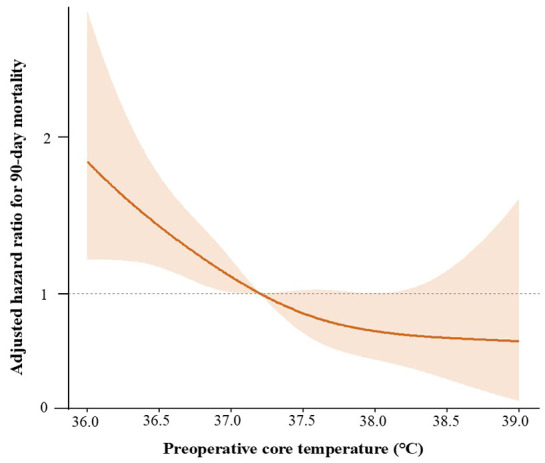
Restricted cubic spline showing the association between preoperative core temperature and 90-day postoperative mortality based on a multivariable Cox proportional hazards model adjusted for age, sex, American Society of Anesthesiologists physical status, diabetes mellitus, total body surface area burned, inhalation injury, albumin, creatinine, vasopressor use, and time from burn injury to surgery. The shaded area represents the 95% confidence interval.

**Figure 4:**
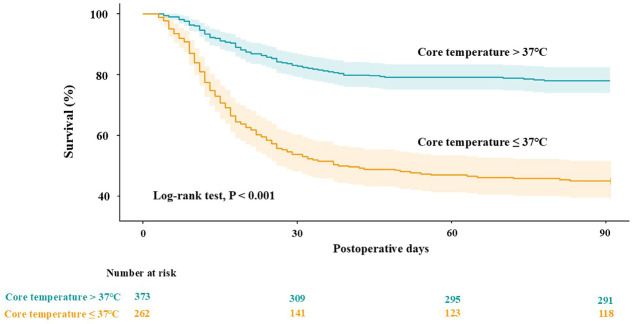
Kaplan–Meier survival curves according to preoperative core temperature. The shaded areas represent the 95% confidence intervals around the survival curves. The cut-off value was determined using receiver operating characteristic curve analysis. The log-rank test was used for group comparison.

Restricted cubic spline analysis demonstrated an inverse linear association between preoperative core temperature and 90-day mortality risk (Figure 3). ROC curve analysis identified 37.0 °C as an exploratory, cohort-specific threshold for risk stratification (area under the curve, 0.733). Kaplan–Meier analysis showed significantly lower 90-day survival in patients with preoperative core temperature ≤37.0 °C than in those with >37.0 °C (45.4% vs. 78.0%, log-rank *p* < 0.001; Figure 4).

In the exploratory, hypothesis-generating cohort, patients with a preoperative core temperature ≤37.0 °C had significantly higher 30-day postoperative complication rates than those with temperatures >37.0 °C. Specifically, the incidences of MACE, bloodstream infections, and CRRT requirement were 24.8% vs. 17.7% (*p* = 0.036), 80.5% vs. 68.4% (*p* = 0.001), and 42.4% vs. 18.0% (*p* < 0.001), respectively (Table 3).

## 4. Discussion

This study demonstrated that preoperative core temperature was significantly associated with 90-day mortality in patients with major burns. Restricted cubic spline analysis further supported an inverse linear association between preoperative core temperature and mortality risk. ROC curve analysis identified 37.0 °C as an exploratory, cohort-specific threshold for risk stratification. A preoperative core temperature ≤37.0 °C was associated with significantly higher rates of 30-day postoperative complications, including MACE, bloodstream infection, and CRRT requirement, compared with higher temperatures in this exploratory, hypothesis-generating cohort. These findings suggest that preoperative core temperature may serve as a clinically relevant marker of physiological vulnerability in this population.

Severe burn injury is characterized by a profound hypermetabolic and inflammatory response accompanied by major alterations in thermoregulation [1,2]. Extensive loss of skin integrity, increased evaporative heat loss, large-volume fluid resuscitation, and cytokine-mediated hypothalamic dysregulation collectively lead to an altered baseline core temperature, even in the absence of infection [2]. As a result, major burn patients exist in a distinct thermophysiological state in which conventional definitions of normothermia may not accurately reflect physiological adequacy [2]. In this context, a core temperature that would be considered normal in other surgical populations may represent a state of relative hypothermia in patients with extensive burns [4,15].

In our Cox proportional hazards regression analysis, preoperative core temperature demonstrated a significant association with 90-day postoperative mortality. Each 1 °C increase in preoperative core temperature was associated with a 36% lower hazard of death (hazard ratio, 0.643), suggesting that even modest differences in thermal status may be prognostically relevant. Restricted cubic spline analysis further demonstrated an inverse linear association between preoperative core temperature and mortality risk. Perioperative hypothermia has long been associated with adverse surgical outcomes, including coagulopathy [16], increased blood loss [17], and wound complications [18], and current guidelines largely define hypothermia as a body temperature below 36 °C [16,19]. Accordingly, most perioperative temperature management strategies focus on maintaining temperature above this threshold [3,20]. However, this definition is derived primarily from general surgical populations and may not be appropriate for patients with major burns, whose baseline thermoregulatory set point is substantially altered. The routine application of a 36 °C threshold may therefore underestimate the physiologic significance of temperature reductions in this population [2]. Burn-specific sepsis criteria adopt higher fever thresholds (e.g., >39 °C) and include hypothermia triggers (<36.5 °C), reflecting the altered inflammatory and thermoregulatory milieu after major burns [21]. However, these diagnostic thresholds may not establish an optimal perioperative or preoperative core temperature target, and reported target ranges vary widely across burn centers [6,7]. This uncertainty highlights the absence of consensus regarding optimal temperature targets in major burns and reinforces the value of preoperative core temperature as a clinically relevant prognostic risk marker [7].

Several studies have reported no significant association between intraoperative hypothermia and mortality in burn patients when active warming strategies are employed. Peng et al. demonstrated that maintaining operating room temperature at 27 °C was sufficient to prevent clinically meaningful deterioration in outcomes, despite frequent episodes of intraoperative hypothermia [11]. In addition, Mai et al. reported that mild postoperative hypothermia was associated with reduced length of hospital stay in adult burn patients, predominantly with minor burns [22]. These studies primarily focused on intraoperative or postoperative temperature and included patients with relatively limited burn severity. Importantly, even in these cohorts, patients who developed hypothermia exhibited significantly lower baseline temperature prior to surgery [11]. Taken together, these observations raise the possibility that preoperative thermal status may capture aspects of physiological vulnerability not fully reflected by intraoperative temperature alone. In patients with major burns, lower preoperative core temperature may reflect reduced capacity to meet the increased metabolic demands imposed by severe injury and may therefore be associated with greater postoperative risk.

We found that lower preoperative core temperature was significantly associated with increased 90-day postoperative mortality in patients with major burns involving TBSA ≥30%. This finding highlights the potential prognostic relevance of preoperative thermal status in patients with severe burn injury. We focused on patients with major burns because hypermetabolic response and thermoregulatory disturbance are generally more pronounced in this population. Previous studies have shown that resting energy expenditure and metabolic demand increase substantially when the TBSA burned exceeds approximately 20%, and this threshold has often been used to define clinically significant systemic burn injury [1,4]. To study a cohort with more severe and sustained physiologic stress while reducing heterogeneity, we restricted the analysis to patients with TBSA ≥30%.

Furthermore, in an exploratory, hypothesis-generating cohort, preoperative core temperature was associated with early postoperative complications, including MACE, bloodstream infection, and CRRT requirement. These complications represent different pathophysiological domains—cardiovascular instability, infectious morbidity, and acute kidney injury—suggesting that preoperative thermal status may reflect a broader physiological vulnerability rather than risk confined to a single organ system. Several mechanisms may explain these associations. Hypothermia is known to increase sympathetic activation, myocardial oxygen demand, and electrical instability, thereby predisposing patients to perioperative cardiac events [23]. In parallel, reduced core temperature impairs innate immune function, alters leukocyte activity, and compromises barrier integrity, which may increase susceptibility to postoperative bloodstream infection [24]. Furthermore, hypothermia induces renal vasoconstriction and reduces renal perfusion, potentially exacerbating ischemic and inflammatory pathways leading to acute kidney injury and subsequent CRRT requirement [25]. These findings suggest that preoperative core temperature may represent more than a simple predictor of mortality and may reflect aspects of early postoperative clinical trajectory in patients with major burns. In the present study, lower preoperative temperature was associated with higher rates of cardiovascular, infectious, and renal complications. Taken together, these results support the possibility that low preoperative temperature reflects reduced physiological reserve and limited capacity to tolerate the metabolic and inflammatory burden of major burn surgery. In this context, preoperative thermal status may serve as an integrative marker for perioperative risk stratification.

Our institutional ICU target temperature range (36.0–38.5 °C) reflects a pragmatic clinical management strategy aimed at preventing overt hypothermia and hyperthermia during burn critical care. In contrast, the 37.0 °C threshold identified in this study was a data-driven, cohort-specific cutoff derived for risk stratification and should not be interpreted as a therapeutic target. Rather, it should be understood as an exploratory prognostic marker indicating that patients with lower preoperative core temperatures, even within the conventional target range, may represent a subgroup with increased physiologic vulnerability.

This study has several limitations. First, its retrospective single-center design limits causal inference and external generalizability. Second, additional markers of preoperative clinical instability—such as active infection or sepsis, shock status, and overt organ failure—were not consistently available in a standardized format across the retrospective study period and therefore could not be reliably incorporated into the primary multivariable model. To partially account for physiologic instability, we instead included surrogate markers available for all patients. However, residual confounding cannot be fully excluded given the retrospective observational design. Third, the strict inclusion and exclusion criteria—limiting the cohort to patients undergoing surgery within 7 days of injury, excluding those with prior positive blood cultures or transfer after initial surgery, and analyzing only the first operation—may have introduced selection bias and limited generalizability. While these criteria improved internal consistency by focusing on the early post-burn period, they may have excluded more complex or severely ill patients, potentially affecting external validity. Fourth, preoperative core temperature was analyzed as a single pre-transfer measurement, and short-term temperature trajectories immediately preceding surgery were not available. Although continuous bladder thermometry was used throughout ICU care, residual variability in temperature dynamics over time cannot be excluded, which may have introduced measurement uncertainty. Finally, the exploratory 37.0 °C threshold derived from this cohort requires external validation in independent populations and should not be interpreted as a definitive therapeutic target. Nevertheless, this study also has several strengths. Patients were treated within a single high-volume specialized burn center using standardized multidisciplinary protocols for surgery, anesthesia, temperature management, and intensive care. This relatively uniform clinical environment reduced treatment heterogeneity and may strengthen internal validity. In addition, the analysis was based on routinely collected objective physiologic, laboratory, and perioperative data from a contemporary cohort of patients with severe burns.

## 5. Conclusions

Lower preoperative core temperature is independently associated with increased 90-day postoperative mortality in patients with major burns. Restricted cubic spline analysis demonstrated an approximately inverse linear association between preoperative core temperature and mortality risk. In this cohort, 37.0 °C served as an exploratory threshold for identifying a higher-risk group, suggesting that conventional definitions of normothermia may not fully reflect physiologic adequacy. Lower preoperative core temperature was also associated with less favorable early postoperative outcomes in the exploratory, hypothesis-generating cohort. These findings support the value of preoperative core temperature as a clinically relevant marker for perioperative risk stratification rather than a causal or therapeutic target, and further external validation and prospective studies are needed.

## Figures and Tables

**Table 1:** Baseline demographic, preoperative laboratory, and intraoperative characteristics.

Variables	Survival Group (*n* = 409)	Non-Survival Group (*n* = 226)	*p*-Value
Age, years	48 (39–57)	54 (46–65)	<0.001
Sex, male	360 (88.0)	185 (81.9)	0.043
Body mass index, kg/m^2^	23.8 (22.0–26.1)	23.8 (22.0–25.7)	0.932
ASA physical status			<0.001
≤2	148 (36.2)	28 (12.4)	
≥3	261 (63.8)	198 (87.6)	
Diabetes mellitus	25 (6.1)	32 (14.2)	0.001
Hypertension	80 (19.6)	57 (25.2)	0.107
Ischemic heart disease	10 (2.4)	11 (4.9)	0.119
Congestive heart failure	3 (0.7)	1 (0.4)	>0.999
Cerebrovascular accidents	12 (2.9)	11 (4.9)	0.267
TBSA burned, %	41.0 (34.0–52.0)	62.5 (46.8–81.0)	<0.001
Presence of inhalation injury	152 (37.2)	140 (61.9)	<0.001
Burn mechanism			<0.001
Flame burn	318 (77.8)	206 (91.2)	
Electrical burn	38 (9.3)	6 (2.7)	
Other burns *	53 (13.0)	14 (6.2)	
Time interval from burn injury to surgery, days	3 (2–5)	3 (2–4)	<0.001
Preoperative laboratory data			
Hemoglobin, g/dL	13.0 (11.2–15.7)	13.3 (10.7–16.5)	0.606
White blood cell, 10^3^/μL	10.8 (6.4–17.1)	16.0 (8.6–26.7)	<0.001
Platelet, 10^3^/μL	165.0 (114.0–236.5)	145.0 (83.0–247.5)	0.018
Albumin, g/dL	2.4 (2.1–2.7)	2.1 (1.8–2.5)	<0.001
Creatinine, mg/dL	0.7 (0.6–0.9)	0.9 (0.7–1.2)	<0.001
Lactic acid, mmol/L	2.7 (1.8–3.9)	4.2 (2.7–6.4)	<0.001
Preoperative core temperature, °C	37.6 (37.0–38.0)	36.8 (36.4–37.3)	<0.001
Intraoperative data			
Operation time, min	90 (65–120)	100 (75–130)	0.031
Usage of vasopressor	151 (36.9)	149 (65.9)	<0.001
Crystalloid amount, mL/kg	17.9 (12.3–26.1)	21.2 (15.4–31.5)	<0.001
Colloid amount, mL/kg	8.9 (6.2–14.3)	10.0 (6.0–14.9)	0.371
RBC transfusion	380 (92.9)	219 (96.9)	0.057
RBC transfusion volume, units	4 (3–6)	5 (4–8)	<0.001

Data are shown as the median (interquartile range) or number of patients (%) as appropriate. * Other burns included scalding burn, contact burn, chemical burn, and spark burn. Preoperative laboratory variables were obtained within 24 h before surgery. Vasopressor use was defined as administration for hypotension (mean arterial pressure <65 mmHg for ≥5 min). No missing data were present for variables included in the primary analysis. ASA, American Society of Anesthesiologists; TBSA burned, total body surface area burned; RBC, red blood cell.

**Table 2:** Univariable and multivariable Cox proportional hazards regression analyses for risk factors associated with 90-day mortality after burn surgery in 635 patients.

Variables	Univariable Analysis		Multivariable Analysis	
	HR (95% CI)	*p*-Value	HR (95% CI)	*p*-Value
Age	1.026 (1.016–1.035)	<0.001	1.045 (1.033–1.058)	<0.001
Male	0.697 (0.497–0.978)	0.037	0.616 (0.429–0.886)	0.009
Body mass index	1.004 (0.966–1.043)	0.840		
ASA physical status				0.004
≤2	1.000		1.000	
≥3	3.333 (2.243–4.952)	<0.001	1.837 (1.210–2.789)	
Diabetes mellitus	1.867 (1.284–2.715)	0.001	1.622 (1.085–2.424)	0.018
Hypertension	1.301 (0.963–1.756)	0.086		
TBSA burned	1.054 (1.047–1.062)	<0.001	1.051 (1.043–1.060)	<0.001
Inhalation injury	2.244 (1.715–2.936)	<0.001	1.569 (1.151–2.140)	0.004
Burn mechanism				
Flame burn	1.000			
Electrical burn	0.297 (0.132–0.668)	0.003	1.039 (0.449–2.405)	0.928
Other burns *	0.408 (0.275–0.813)	0.007	0.735 (0.412–1.311)	0.297
Time interval from burn injury to surgery	0.884 (0.827–0.945)	<0.001	1.049 (0.981–1.122)	0.161
Hemoglobin	0.998 (0.975–1.022)	0.871		
White blood cell	1.032 (1.023–1.042)	<0.001	1.010 (0.997–1.024)	0.141
Platelet	1.000 (0.999–1.001)	0.998		
Albumin	0.446 (0.351–0.567)	<0.001	0.763 (0.599–0.972)	0.029
Creatinine	2.046 (1.695–2.469)	<0.001	1.613 (1.288–2.020)	<0.001
Lactic acid	1.007 (0.999–1.015)	0.100		
Preoperative core temperature	0.359 (0.293–0.440)	<0.001	0.643 (0.513–0.805)	<0.001
Operation time	1.004 (1.001–1.007)	0.009	0.999 (0.997–1.002)	0.471
Usage of vasopressor	2.647 (2.010–3.487)	<0.001	1.359 (1.009–1.830)	0.043
Crystalloid amount	1.017 (1.009–1.025)	<0.001	0.995 (0.985–1.005)	0.366
RBC transfusion	2.162 (1.019–4.589)	0.045	1.751 (0.792–3.875)	0.167

* Other burns included scalding burn, contact burn, chemical burn, and spark burn. HR, hazard ratio; CI, confidence interval; ASA, American Society of Anesthesiologists; TBSA burned, total body surface area burned; RBC, red blood cell.

**Table 3:** 30-day postoperative complications according to preoperative core temperature.

Variables	Core Temperature >37 °C (*n* = 373)	Core Temperature ≤37 °C (*n* = 262)	*p*-Value
MACE	66 (17.7)	65 (24.8)	0.036
Bloodstream infection	255 (68.4)	211 (80.5)	0.001
CRRT	67 (18.0)	111 (42.4)	<0.001

Data are shown as the number of patients (%). MACE, major adverse cardiovascular events; CRRT, continuous renal replacement therapy.

## Data Availability

The data presented in this study are available on request from the corresponding author. Data are not publicly available due to ethical considerations.

## Abbreviations

The following abbreviations are used in this manuscript:

MACE	major adverse cardiovascular events
CRRT	continuous renal replacement therapy
ICU	intensive care unit
TBSA	total body surface area
ASA	American Society of Anesthesiologists
ROC	receiver operating characteristic
